# Plasticity of Intestinal Epithelium: Stem Cell Niches and Regulatory Signals

**DOI:** 10.3390/ijms22010357

**Published:** 2020-12-31

**Authors:** Ken Kurokawa, Yoku Hayakawa, Kazuhiko Koike

**Affiliations:** Department of Gastroenterology, Graduate School of Medicine, The University of Tokyo, Tokyo 113-8655, Japan; ken.kurokawa178@gmail.com (K.K.); kkoike-tky@umin.ac.jp (K.K.)

**Keywords:** intestinal stem cells (ISCs), dedifferentiation, Wnt, Notch, cytokines

## Abstract

The discovery of Lgr5+ intestinal stem cells (ISCs) triggered a breakthrough in the field of ISC research. Lgr5+ ISCs maintain the homeostasis of the intestinal epithelium in the steady state, while these cells are susceptible to epithelial damage induced by chemicals, pathogens, or irradiation. During the regeneration process of the intestinal epithelium, more quiescent +4 stem cells and short-lived transit-amplifying (TA) progenitor cells residing above Lgr5+ ISCs undergo dedifferentiation and act as stem-like cells. In addition, several recent reports have shown that a subset of terminally differentiated cells, including Paneth cells, tuft cells, or enteroendocrine cells, may also have some degree of plasticity in specific situations. The function of ISCs is maintained by the neighboring stem cell niches, which strictly regulate the key signal pathways in ISCs. In addition, various inflammatory cytokines play critical roles in intestinal regeneration and stem cell functions following epithelial injury. Here, we summarize the current understanding of ISCs and their niches, review recent findings regarding cellular plasticity and its regulatory mechanism, and discuss how inflammatory cytokines contribute to epithelial regeneration.

## 1. Discovery of Lgr5+ Stem Cells and Their Niche

The intestinal epithelium consists of the villus part, which faces the lumen and plays an important role in food digestion and absorption, and the crypt part, which is located between the villus as an invaginated form and acts as a source of epithelial turnover. Intestinal stem cells (ISCs) have been thought to reside within the crypts and continuously supply their daughter differentiated cells from crypt to villi. While the majority of the differentiated daughter cells turn over within 3–5 days, ISCs are long-lived and can self-renew [1]. Earlier studies revealed that there are label-retaining cells located at the +4 position within the proliferating zone of crypts, which were initially thought to be ISCs [2]. However, recent studies using the lineage tracing method identified crypt base columnar (CBC) cells as rapidly cycling, self-renewing ISCs residing at the +1 to +3 position of the crypt [3].

Leucine-rich repeat-containing G protein-coupled receptor 5 (Lgr5) is a receptor of R-spondin, and its binding strengthens Wnt signaling through the stabilization of β-catenin [4]. Lgr5 was found to be specifically expressed in CBC cells [3]. Lineage tracing experiments using Lgr5-CreERT mice showed that Lgr5+ CBC cells have the capacity of long-term self-renewal and differentiation into multiple cell types, indicating that Lgr5+ CBC cells have a stem cell function.

Several pathways are strongly activated in ISCs to maintain their stem cell activity. Wnt signaling, transduced through β-catenin/transcription factor 4 (Tcf4), is essential for intestinal development during the embryonic stage and plays a critical role in mucosal homeostasis and stem cell functions in the adult intestinal epithelium [5]. The Notch receptor Notch1 is expressed in Lgr5+ CBC cells, and Notch signaling is required for stem cell maintenance [6,7]. Inhibition of notch signaling induces rapid stem cell loss, with reduced proliferation and apoptosis, and promotes differentiation into secretory cell lineages. BMP signaling suppressed Wnt signaling to ensure a balanced control of ISC self-renewal, and conditional inactivation of Bmpr1a in mice increased the stem and progenitor cell populations, eventually inducing intestinal polyposis [8].

**Figure 1:**
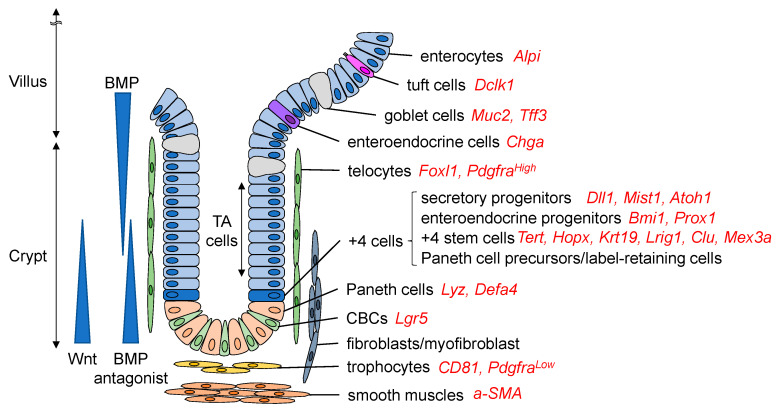
Intestinal stem cells and their niches. Lgr5+ crypt base columnar (CBC) cells reside at the crypt base and continuously supply their daughter cells from crypt to villi. Paneth cells, interspersed between Lgr5+ CBC cells, can act as a stem cell niche to maintain the stem cell functions of CBC cells. There is another stem cell pool, the +4 cells, which contain quiescent +4 stem cells, Paneth cell precursors, and label-retaining cells. Transit-amplifying (TA) cells include more differentiated and actively proliferating but relatively short-lived cell populations, including both secretory and absorptive progenitors, which can give rise to stem-like cells following stem cell damage. Secretory progenitors are differentiated into Paneth cells, goblet cells, tuft cells, and enteroendocrine cells, while absorptive progenitors enterocytes. The stromal cells surrounding the crypt region contribute to the stem cell niche and are classified into several subsets, such as fibroblasts, myofibroblasts, telocytes, and trophocytes, all of which express and secrete stem cell niche factors. Representative markers of each cell are shown in red.

**Figure 2:**
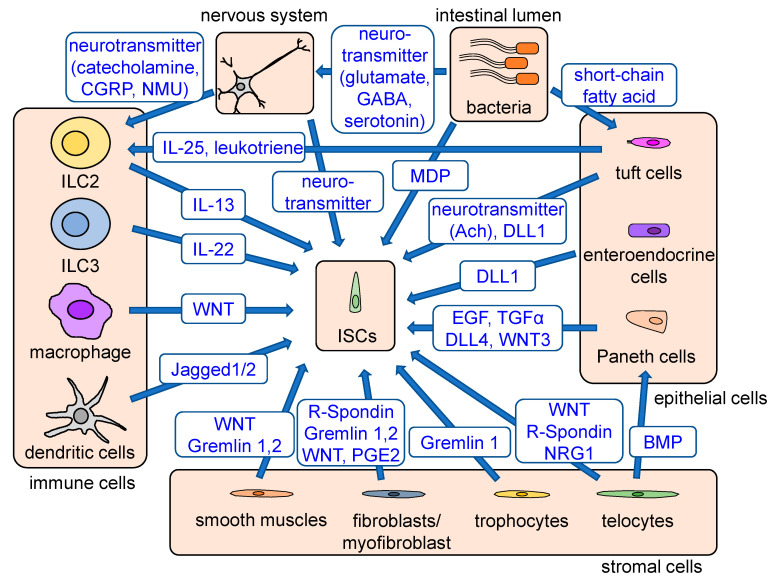
Stem cell niches and regulatory signals. Stem cell niches consist of epithelial cells, stromal cells, immune cells, bacteria, the nervous system, and other signals. Paneth cells, interspersed between Lgr5+ CBC cells, secrete EGF, TGFα, DLL4, and WNT3 and maintain stem cell functions. When Paneth cells are ablated, tuft and enteroendocrine cells act as a complementary source of Notch signaling. Fibroblasts maintain stem cell functions by producing WNT, R-spondin, gremlin, and PGE2. Foxl1+Pdgfra^High^ telocytes provide Wnt, R-spondin, and BMP, while CD81+Pdgfra^Low^ trophocytes at the crypt bottom secrete BMP antagonists and gremlin 1 to strengthen WNT signaling. WNT and BMP antagonists are also secreted from smooth muscles. The cytokines secreted from immune cells, including Wnt-producing macrophages, Jagged-producing dendritic cells, IL13-producing ILC2s, and IL22-producing ILC3s, have an important role in stem cell regulation and epithelial regeneration. Enteric bacteria either directly or indirectly regulate stem cell functions via production of MDP, which supports ISC survival and activation of the tuft cell–ILC2 immune circuit through their metabolites. Tuft cells are involved in the activation of ILC2s via production of IL-25 and leukotrienes. Enteric nerves and tuft cells support ISC functions via production of acetylcholine, and various neuron-derived products can stimulate ILC2s, which support ISC function. Major signals provided from stem cell niches are shown in blue.

Intestinal stem cells are supported by the surrounding niche cells to maintain their proliferative, self-renewing ability. Multiple cell types contribute to the stem cell niche (Figure 1). Paneth cells, interspersed between Lgr5+ CBC cells, play a role in mucosal immune defense and act as niche cells by secreting several growth factors, such as Egf, Tgf-a, Wnt3, and Notch ligand Dll4, all of which activate critical signals for stem cell maintenance [9]. Nevertheless, even if Paneth cells are ablated, intestinal homeostasis is not impaired, as other niche cells, such as tuft cells, enteroendocrine cells, and stromal cells, can compensate for the loss of Paneth cells [9,10,11]. The stromal cells play essential roles as stem cell niches. The major intestinal stromal subsets are classified as fibroblasts, a-smooth muscle actin (a-SMA)-expressing myofibroblasts, and perivascular pericytes [12,13]. Studies using in situ hybridization revealed that many Wnt ligands, such as Wnt-2b, Wnt-4, Wnt-5a, and Wnt-5b, as well as R-spondins, are expressed in the stroma to support ISCs (Figure 2) [14,15]. Foxl1+ telocytes, a small subset of fibroblasts, are one of the important sources of Wnts, and the ablation of telocytes inhibited the proliferation of ISCs, which was associated with a loss of active Wnt signaling [16,17]. Recent studies have revealed more detailed subsets among these stromal cell types, showing that Foxl1+Pdgfra^High^ telocytes secrete Wnt ligands, R-spondins, and BMPs, while CD81+Pdgfra^Low^ trophocytes at the crypt bottom secrete BMP antagonists and gremlin 1 to maintain WNT signaling [18,19]. 

The EGFR pathway is activated in the stem and progenitor cells, and multiple EGFR ligands mediate this pathway. As mentioned above, Paneth cells are likely involved in this signaling, but more recent studies have identified neuregulin 1 (NRG1) as a predominant EGFR ligand that activates ISCs during epithelial repair and development [20,21]. NRG1 is upregulated in the stromal compartment of the regenerating intestine and is indispensable for tissue repair. NRG1, but not EGF, increases cellular diversity in cultured enteroids, highlighting the importance of NRG1 as a stem cell niche factor. BMP antagonists, including gremlin 1, gremlin 2, and chordin-like 1, are also expressed by intestinal pericryptal myofibroblasts and smooth muscle cells at the colon crypt [22]. Other reports showed that the mesenchymal cells express prostaglandin E2 (PGE2) to promote epithelial regeneration through the activation of YAP signaling [23,24]. 

Bacterial pathogens in the intestinal lumen also, in many ways, contribute to the activity of ISCs. ISCs express a high level of an innate immune receptor, nucleotide-binding oligomerization domain-containing protein 2 (NOD2) receptor, and can recognize bacteria-derived products through NOD2. A common peptidoglycan motif, muramyl dipeptide (MDP), supports ISC survival through the clearance of the mitochondrial reactive oxygen species (ROS) within ISCs via mitophagy in a NOD2-dependent manner [25]. Another receptor for bacterial pathogens, toll-like receptor 4 (TLR4), also plays an important role in intestinal homeostasis and the protection against epithelial damages. Tlr4^–/–^ mice as well as germ-free, wild-type mice were more susceptible to epithelial damage induced by DSS treatment [26]. Subsequent studies have suggested that the proliferation of ISCs appears to be mediated at least in part by TLR4, as well as downstream expression of the specific microRNA such as miR-375-3p [27,28].

Recent studies have suggested that gut microbiota modulate not only gut homeostasis but also functions and diseases in the distant organs, including the central nervous system (CNS), liver, pancreas, and skin [29]. In particular, the gut–brain axis has been shown to be involved in various diseases through bidirectional communication between the intestine and the central nervous system, which is largely mediated by the gut microbiome. Several mechanisms have been suggested concerning the gut–brain axis. First, the microbiota-derived metabolites and neurostimulatory peptides, such as glutamate, gamma-aminobutyric acid (GABA), and serotonin, can act as neurotransmitters to stimulate the CNS, as well as the enteric nervous system [30]. The bacterial metabolites are also related to other extra-intestinal disorders, including non-alcoholic steatohepatitis [31]. Second, specific cytokines and immune cells can be activated in the intestinal bacteria, and they play an important role in ISC regulation and affect the gut–brain axis. For example, gut microbiota are associated with the development and severity of multiple sclerosis [32], since autoreactive T cells, which respond to myelin oligodendrocyte glycoprotein (MOG), can be activated by specific intestinal bacterial species, such as the family of *Erysipelotrichaceae* and a strain of *Lactobacillus reuteri*. In addition to multiple sclerosis, it has been reported that there is a strong association between gut dysbiosis and other neurological and psychological disorders, including Parkinson’s disease, amyotrophic lateral sclerosis, Alzheimer’s disease, and autism spectrum disorder. Teratani et al. recently proposed a liver–brain–gut axis, in which vagus nerve signals in the liver mediate the differentiation of peripheral regulatory T cells (pTreg cells) in the gut and subsequent intestinal inflammation [33]. As surgical resection of the hepatic vagal sensory afferent nerves reduced pTreg cells and increased susceptibility to colitis, the liver seems to be an important relay point within the gut–brain axis.

The nervous system is considered a part of the stem cell niche. Acetylcholine (ACh), a major neurotransmitter in the enteric nervous system, is associated with multiple functions, including movement, secretion, and endocrine [34]. In addition, as the nonselective blockade of muscarinic receptors using scopolamine reduced both the number and activity of Lgr5+ ISCs [35,36,37], ACh-producing nerves play a role in the ISC niche. Interestingly, epithelial tuft cells, which usually express a specific marker, Dclk1, can act as a source of ACh [38], and the expansion of Dclk1+ tuft cells was observed following the administration of scopolamine via a negative-feedback loop. Although a muscarinic receptor, M3R, was expressed in several cell types in the intestine, Prox1+ enteroendocrine cell-specific ablation of M3R induced robust tuft cell expansion, suggesting that the Prox1+ cells monitor and sustain the murine intestinal epithelial cholinergic niche.

## 2. Plasticity within Intestinal Stem and Progenitor Cells

Recently, it has been suggested that short-lived progenitors and even a subset of mature cells can dedifferentiate and function as an alternative source of ISCs during inflammation and regeneration. The ablation of Lgr5+ CBC cells using a diphtheria toxin receptor gene did not affect the homeostasis of the intestinal epithelium in mice, suggesting the presence of another reserve stem cell pool [39]. The currently accepted theory is that there are two major, functionally distinct populations of stem cells: the Lgr5+ CBC cells, which divide rapidly at the crypt base, and the more quiescent +4 stem cells, which reside at the +4 position above CBC cells. The +4 stem cells are marked by unique markers, such as Bmi1, Tert, Hopx, Krt19, Clu, Mex3a, or Lrig1 [39,40,41,42,43,44,45,46], and can self-renew and modestly trace the entire villus crypt units in the normal state, but become more active following epithelial injury [47]. While Lgr5+ stem cells are susceptible to various types of epithelial injury induced by irradiation [41,48,49,50], chemicals [51,52,53], and pathogens [54,55], and easily undergo apoptosis, the +4 stem cells are resistant to such epithelial injury and serve as a reserve stem cell population [39,48]. Following the loss of Lgr5+ cells, the +4 reserve stem cells interconvert to Lgr5+ ISCs and act as a major source of cell supply. At this stage, the re-emerged Lgr5+ stem cell pool is indispensable for epithelial regeneration [56].

Apoptosis in Lgr5+ ISCs occurs predominantly through the p53/PUMA-dependent pathway, and blockade of this pathway prolongs the survival of Lgr5+ cells and promotes epithelial regeneration [52,57]. Apoptosis in +4 stem cells can be induced by tamoxifen, a reagent that excites the Cre–LoxP gene recombination system in a Bcl2-dependent manner [58], and this effect might influence the frequency of lineage-tracing events from Lgr5+ and +4 ISCs. Activation of the CreERT system in the intestinal epithelium impairs stem cell functions by causing genome toxicity [59], suggesting that previous findings using the CreERT-dependent lineage-tracing system may need to be carefully reinterpreted.

Transcriptome analyses at the single-cell level revealed that at least a subset of +4 ISCs, including Bmi1+ cells, may belong to the enteroendocrine lineage [60]. Another group reported that Bmi1+ cells expressed the enteroendocrine cell-related genes, including Prox1, and lineage tracing showed that Prox1+ cells maintained both the homeostasis and regeneration of the intestinal epithelium [61]. Single-cell mRNA-seq revealed that the Prox1+ cells consist of two subgroups: enteroendocrine and tuft cell lineages [37,61]. In addition, CD69+ and CD274+ goblet cell precursors can dedifferentiate into Lgr5+ stem cells in response to stem cell ablation [62]. Secretory precursors that express Dll1 or Atoh1 [63] also act as a reserve stem cell pool and can give rise to stem-like cells after epithelial injury [64,65]. Our group recently identified Bhlha15+ (also known as Mist1+) secretory precursors that can dedifferentiate into stem-like cells in the small intestine and the colon. Dedifferentiation from Bhlha15+ progenitors occurs in a Notch-dependent manner in the small intestine, and in a YAP-dependent manner in the colon [53]. Finally, in addition to the secretory precursors, Alpi+ enterocyte-lineage progenitors also had the capacity to replace lost stem cells during intestinal regeneration [66]. 

The dedifferentiation and interconversion from the progenitors to the stem cells are controlled by multiple mechanisms, including epigenetic modification and Wnt, Notch, and Ascl2-dependent signaling. Jadhav et al. identified that thousands of cis elements that control lineage-restricted gene expression are selectively open in secretory cells and the accessibility dynamically converts toward that of Lgr5+ stem cells in response to the Lgr5+ stem cell loss [62]. As Wnt ligands are required for organoid growth from Dll1+ secretory progenitors, the importance of Wnt signaling in dedifferentiation has been suggested [63]. Systemic inhibition of Wnt secretion with porcupine inhibitors impaired intestinal homeostasis after radiation injury [15]. During tissue damage and regeneration, inflammatory cells such as macrophages serve as an important source of Wnt ligands [67]. 

Likewise, inhibition of Notch signaling using a γ-secretase inhibitor resulted in impaired proliferation and the loss of the regenerative response within the epithelial layer after DSS treatment [68], and the deletion of the Notch 1 or Notch 2 receptor reduced epithelial proliferation and impaired crypt regeneration after radiation [69], suggesting the importance of Notch signaling after injury. In inflammatory conditions, tissue-resident dendritic cells may serve as niche cells that activate Notch signaling in ISCs [70]. Ascl2, a transcription factor that cooperates with β-catenin/Tcf4 and controls the stem-cell-specific subset of intestinal Wnt target genes [71], has also been suggested as an essential factor for stem cell interconversion. Ascl2 activates IL-11RA signaling, which promotes a regenerative response in the intestinal epithelium [72].

## 3. Plasticity in Mature Cell Types and Potential as an Origin of Cancer

In addition to the progenitors, several reports have shown that a few mature cells can also dedifferentiate and give rise to stem cells. Studies using Paneth-cell-labeled transgenic mice suggested that mature Paneth cells may be capable of dedifferentiating in response to irradiation and acting as stem-like cells [73]. Activation of Notch signaling and SCF signaling appears to be required for the dedifferentiation from Paneth cells [51]. Phosphatidylinositol 3-kinase (PI3K)/Akt and Wnt signaling are also activated during the dedifferentiation of Paneth cells following chemically induced intestinal injury [74]. 

In the colon, more differentiated Krt20+ surface enterocytes can contribute to the epithelial regeneration in response to the loss of Lgr5+ stem cells during DSS-induced injury [75]. During colonic regeneration, there is an upregulation of R-spondin 3 in the stroma, which appears necessary to enhance Wnt signaling and dedifferentiation from differentiated Krt20+ cells.

Plasticity in mature cell types may potentially initiate carcinogenesis. Simultaneous Notch activation with the loss of Apc in Paneth cells induces tumor formation in the intestine, suggesting the possibility that Paneth cells may serve as the origin of intestinal tumors under specific conditions [51,73,74]. Consistently, aberrant appearance of Paneth cells is frequently observed in intestinal epithelial lesions, including colorectal cancer [76]. Tuft cells have been suggested as another source of mature cell-derived tumorigenesis. It was reported that Dclk1+ tuft cells [77] or IL17RB+ tuft-like cells [78] act as stem-like cells in the established intestinal tumors. Similarly, Apc deletion in Dclk1+ tuft cells generates colonic tumors in the setting of additional DSS-induced inflammation, while tumor formation from Dclk1-expressing cells was not observed in the steady state [79]. These results suggest that intestinal tuft cells, some of which appear to be extremely long-lived, can act as colon cancer-initiating cells following acute injury. However, it remains unclear as to what factors are a key trigger for tuft cell-derived tumor formation. As the activation of NF-κB signaling is reportedly required for dedifferentiation and tumor formation from a non-stem cell population, several key pathways such as NF-κB signaling would probably regulate tuft cell activity during inflammation, and this point needs to be elucidated in future studies [80].

## 4. Role of Inflammatory Signals in Cellular Plasticity and Homeostasis

Recently, it has been elucidated that inflammatory cytokines play an important role in stem cell regulation, especially during tissue injury and regeneration. Such cytokines are produced by multiple immune cell types, including innate lymphoid cells (ILCs), macrophages, and dendritic cells. ILCs consist of three subtypes, ILC1s, ILC2, and ILC3, and are differentiated from common lymphoid progenitors to secrete specific effector molecules such as interleukin (IL) [81,82] (Table 1). ILC1 secretes interferon-γ and tumor necrosis factor and mainly contributes to the protection against intracellular pathogens, while ILC2 and ILC3 contribute to the resolution of inflammation and regeneration, in addition to their role in initiating acute inflammatory responses [82].

ILC2s are involved in type 2 immune responses, which are mediated by type 2 cytokines, including IL33, IL25, IL13, and IL5. Among these cytokines, IL-25 was found to be secreted specifically from tuft cells, and tuft-cell-derived IL-25 activated ILC2s via the IL-17RB receptor. Activated ILC2s secreted IL-13 and stimulated ISCs to promote tuft and goblet cell differentiation [83]. During helminth or protist infection, the tuft cells dramatically increased, and the activated tuft cell–ILC2–ISC circuit contributed to the clearance of the infected pathogens [84]. More recently, it was reported that circular RNA circPan3 binds to mRNA, encoding the IL-13 receptor subunit IL-13Rα1, and increases its stability to induce the expression of IL-13Rα1 in ISCs [85]. The circPan3-dependent IL13Ra1 signaling appears to be essential for self-renewal in Lgr5+ ISCs and regeneration of the intestinal epithelium. Thus, ILC2 and type 2 immune cytokines play critical roles in ISC functions and mucosal homeostasis. ILC2s also contribute to the progression of gastric and biliary cancers, possibly related to the secretion of Wnt5a or IL-33 [86,87]. In addition, ILC2s are closely related to nervous signaling and activated by various neurotransmitters and neuron-derived products, including neuromedin U (NMU) [88,89,90], alpha-calcitonin gene-related peptide (a-CGRP) [91,92,93], and adrenaline [94,95]. NMU, a ligand of a neuropeptide receptor Nmur1, is selectively expressed in ILC2s, and the activation of NMU–Nmur1 signaling strongly induces the production of innate inflammatory and tissue repair cytokines. a-CGRP, a 37 amino acid neuropeptide produced by alternative splicing of the calcitonin gene, is highly expressed in a subset of ILC2s, which preferentially express IL-5 after infection, and negatively modulates the production of type 2 cytokines of ILC2s. In addition, ILC2s also express the b2-adrenergic receptor and colocalize with adrenergic neurons in the intestine. The adrenergic signaling suppresses the ILC2 responses and reduces inflammation. These findings again highlight the importance of the gut–brain axis in gut immunity and pathogenesis.

IL-22, a member of the IL-10 family, is predominantly secreted from ILC3s, and the expression of IL-22 receptors (IL-22R) is restricted mainly to the TA cells in the intestine [96]. The IL-22–IL22R interaction contributes to the mucosal healing by inducing epithelial proliferation and regeneration after damage [82]. Lindemans et al. showed that IL-22 induced the phosphorylation of STAT3 in Lgr5+ ISCs, and the activated STAT3 was crucial for organoid formation and IL-22-mediated tissue regeneration [97]. More recently, two studies have reported that IL-22 interacts with transit-amplifying cells in addition to ISCs [98,99]. Zwarycz et al. showed that in the in vitro ileal organoid model, the stimulation with IL-22 increased the size of organoids but decreased the organoid survival, accompanied with reduced expression of ISCs markers (Lgr5, Olmf4) and Wnt and Notch signaling [98]. They also reported that the proliferating cells increased in the TA zone without affecting the number of ISCs in IL-22-trangenic mice. Zha et al. also showed that IL-22 markedly reduced the number of Lgr5+ ISCs using Lgr5 reporter mice, but increased epithelial proliferation and markers of the TA zone [99]. In addition, it was reported that IL-22 is produced by recipient ILC3s, which can persist after bone marrow transplant (BMT) and protect ICSs from immune-mediated tissue damage by graft versus host disease (GVHD) [100]. ILC3s also amplify the YAP1 signaling in intestinal crypt cells and contribute to the regeneration of the intestinal epithelium in an IL-22-independent manner [101].

Another class of cytokines, IL-6, is one of the major proinflammatory cytokines, and it influences multiple processes, including inflammation, cell proliferation, and survival [102]. Although IL-6 family members were traditionally thought to activate JAK–STAT3 signaling via the common co-receptor gp130, Taniguchi et al. reported that IL-6 contributed to epithelial regeneration through gp130–Src–YAP–Notch signaling [103]. Transgenic mice that expressed an activated form of gp130 (gp130^Act^) enhanced YAP and Notch signaling and caused aberrant proliferation of the intestinal epithelium, while Notch or YAP inhibition using inhibitors or gp130^Act^;YAP^Fl/Fl^ mice restored intestinal epithelial homeostasis. The gp130^Act^ mice also exhibited less severe colitis and weight loss than wild-type mice after DSS-induced injury. In addition, autocrine IL-6 signaling also contributed to crypt homeostasis through the Paneth cells and the Wnt signaling pathway [104]. Exogenous IL-6 promoted crypt organoid proliferation in vitro and increased ISCs through JAK–STAT3 and Wnt signaling in Paneth cells, while inhibition of IL-6 signaling reduced organoid proliferation in vitro and the number of Lgr5+ ISCs and Paneth cells in vivo. As IL-11, which also binds to the gp130 co-receptor, may be involved in stem cell interconversion, as mentioned above, signals mediated by the gp130 receptor seem to play critical roles in maintaining intestinal homeostasis, particularly during injury. 

It has been reported that interferon (IFN)-γ is a principal mediator of immune-mediated damage responses in ISCs, especially after BMT [105]. Although few T cells can be found near the ISCs in a normal intestine, donor T cells infiltrate the intestinal crypt following BMT. In this setting, IFN-γ secreted from the T cells can directly target ISCs to induce their apoptosis through JAK–STAT signaling. In addition, IFN signaling also influences the regenerative capacity of ISCs [106]. Interferon regulatory factor 2 (IRF2), which is a negative regulator of IFN signaling, appears to be essential for intestinal regeneration after 5-fluorouracil-induced damage, based on the observations in Irf2^–/–^ mice. Lgr5+ ISCs significantly reduced and instead immature Paneth cells increased in Irf2^–/–^ intestines, indicating that excessive IFN signaling directs ISCs towards a secretory-cell fate. Moreover, Th17-associated IL-17A, in addition to Th1-associated IFN-γ, also has critical effects on tissue damage and regeneration by mediating C-X-C motif ligand 10/interferon-inducible protein 10 (CXCL10/IP10) signaling [107]. 

## 5. Conclusions

ISCs, including Lgr5+ CBC cells and +4 stem cells, cooperatively maintain intestinal homeostasis. In addition, more differentiated progenitors and specific cell types contribute to epithelial regeneration via dedifferentiation. The stem cell niche regulates stem cell functions in the normal state and boosts cellular plasticity and dedifferentiation during injury. In particular, immune cells, such as ILCs, T cells, dendritic cells, and macrophages, as well as the nervous system, play important roles in the homeostasis of ISCs and intestinal regeneration. Understanding the complex, stratified regulatory systems for ISC maintenance will be useful for innovative therapy for intestinal injury, inflammatory bowel disease, and intestinal neoplasm in the near future.

## Figures and Tables

**Table 1:** ILCs and regulatory cytokines.

Groups	Subgroups	Factors Required for Differentiation of ILCs	Cytokines Stimulating ILCs	Cytokines Secreted from Activated ILCs	Functions
ILC1s	NK cells	T-bet, eomes	IL-12, IL-18	IFN-γ	Early immune responses against viruses Immune responses against cancer cells
	ILC1 cells	T-bet	IL-12, IL-18	IFN-γ, TNF	Protection against intracellular pathogens
ILC2s	ILC2 cells	GATA3, RORα	IL-25, IL-33, TSLP	IL-4, IL-5, IL-13	Protection against helminth parasites Promotion of mucus production from goblet cells Resolution of inflammation and regeneration
ILC3s	LTi cells	RORγt	IL-1β, IL-23	IL-17, IL-22	Formation of lymph nodes during embryogenesis
	ILC3 cells	RORγt	IL-1β, IL-23	IL-17, IL-22, IFN-γ	Protection against fungi and extracellular bacteria Promotion of antimicrobial peptide from Paneth cells Resolution of inflammation and regeneration

Abbreviations: eomes, eomesodermin; GATA, GATA-binding protein; IFN, interferon; IL, interleukin; ILCs, innate lymphoid cells; LTi cells, lymphoid tissue inducer cells; NK cells, natural killer cells; ROR, retinoic acid receptor-related orphan receptor; TSLP, thymic stromal lymphopoietin.
